# A host-directed macrocyclic peptide therapeutic for MDR gram negative bacterial infections

**DOI:** 10.1038/s41598-021-02619-y

**Published:** 2021-12-06

**Authors:** Justin B. Schaal, Yoshihiro Eriguchi, Dat Q. Tran, Patti A. Tran, Chase Hawes, Anthony E. Cabebe, Kaitlyn Pike, Katie Trinh, André J. Ouellette, Michael E. Selsted

**Affiliations:** 1 Department of Pathology and Laboratory Medicine, Keck School of Medicine, University of Southern California, Los Angeles, CA USA; 2 Oryn Therapeutics, Vacaville, CA USA; 3 Norris Comprehensive Cancer Center of the University of Southern California, Los Angeles, CA USA; 4 Present Address: Department of Clinical Immunology and Rheumatology/Infectious Disease, Kyushu University Hospital, Fukuoka, Japan; 5 Present Address: Department of Medicine and Biosystemic Science, Kyushu University Graduate School of Medical Science, Fukuoka, Japan

**Keywords:** Infection, Inflammation, Antimicrobials, Bacterial infection

## Abstract

The emergence of infections by carbapenem resistant Enterobacteriaceae (CRE) pathogens has created an urgent public health threat, as carbapenems are among the drugs of last resort for infections caused by a growing fraction of multi-drug resistant (MDR) bacteria. There is global consensus that new preventive and therapeutic strategies are urgently needed to combat the growing problem of MDR bacterial infections. Here, we report on the efficacy of a novel macrocyclic peptide, minimized theta-defensin (MTD)-12813 in CRE sepsis. MTD12813 is a theta-defensin inspired cyclic peptide that is highly effective against CRE pathogens *K. pneumoniae* and *E. coli *in vivo. In mouse septicemia models, single dose administration of MTD12813 significantly enhanced survival by promoting rapid host-mediated bacterial clearance and by modulating pathologic cytokine responses, restoring immune homeostasis, and preventing lethal septic shock. The peptide lacks direct antibacterial activity in the presence of mouse serum or in peritoneal fluid, further evidence for its indirect antibacterial mode of action. MTD12813 is highly stable in biological matrices, resistant to bacterial proteases, and nontoxic to mice at dose levels 100 times the therapeutic dose level, properties which support further development of the peptide as a first in class anti-infective therapeutic.

## Introduction

Infections caused by CRE represent a global threat to human health. The Centers for Disease Control and Prevention (CDC) and the World Health Organization (WHO) categorize CRE infections as a major and urgent threat to public health. Resistance of CRE to carbapenem antibiotics leaves few treatment options other than colistin and polymyxin B, both of which have limited use because of their toxicities^1,2^. Among Enterobacteriaceae, infections by *Klebsiella pneumoniae* and *Escherichia coli* cause an estimated 140,000 nosocomial infections per year in the United States alone, and an increasing fraction are carbapenem resistant^1^. Of the β-lactam antibiotics, carbapenems have the broadest activity spectrum and greatest potency against Gram negative bacteria^3–5^. However, the incidence of CRE infections continues to rise in health care settings and in the community^4,5^, underscoring the need for new therapeutic countermeasures. In this regard, most efforts have focused on developing new carbapenem/β-lactam-based drugs, some which are combined with β-lactamase inhibitors^3^. Carbapenem resistance is multifactorial, involving acquisition of new or mutant β-lactamases, efflux pumps, loss of outer membrane porins, and alterations of penicillin binding proteins^6,7^, and combinations of these resistance mechanisms broaden resistance to carbapenems and other antibiotics by CRE^5^, especially in *K. pneumoniae*.

Here we report a novel therapeutic strategy employing a macrocyclic peptide that is highly effective in a mouse model of CRE bacteremic sepsis. The peptide, MTD12813, acts by promoting host-mediated clearance of the pathogen by stimulating phagocytosis, while homeostatically modulating dysregulated systemic inflammation.

**Figure 1:**
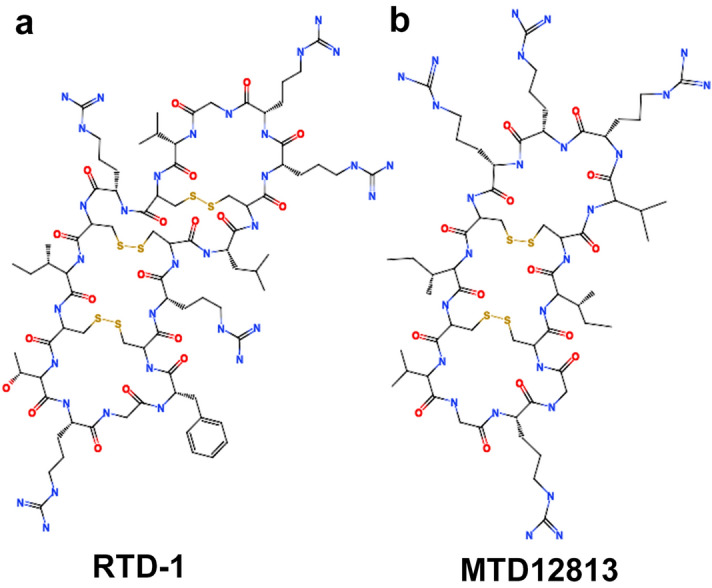
Natural Rhesus theta defensin 1 (RTD-1) and bioinspired MTD12813. Molecular structures of 18-amino acid RTD-1 (**a**) and 14-amino acid MTD12813 (**b**).

MTD12813 is a 14-amino acid macrocyclic peptide containing two disulfide bonds (Fig. 1) that possesses features of naturally occurring macrocyclic θ-defensins expressed uniquely in myeloid and epithelial cells of Old World monkeys (OWM, e.g., rhesus macaques, baboons, vervets, cynomolgus monkeys)^8,9^ but not in other primates or non-primates. All known natural θ-defensins are 18-amino acid cyclic peptides containing three disulfides (Fig. 1)^8–10^ which confers remarkable stability in vitro and in vivo^11–14^. Studies show that θ-defensins possess potent antimicrobial activities in vitro and unique anti-infective and immunoregulatory properties in vivo. The prototype rhesus macaque θ-defensin^14^ RTD-1 (Fig. 1a) is effective in preclinical models of severe sepsis^11^, systemic candidiasis^15^, SARS-CoV-1 infection^16^, chronic *P. aeruginosa* infection in cystic fibrosis^12^, endotoxin-induced lung injury^17^, and autoimmune arthritis^18^. We and others have hypothesized that θ-defensins contribute to the unique host defense and immunomodulatory responses of OWM^19^, primates that, compared to humans and other hominins, are intrinsically resistant to bacterial infections, endotoxemia, and sepsis^20–23^. In sepsis models, θ-defensins facilitate host-mediated clearance of bacterial and fungal pathogens while moderating cytokine-driven immunopathology^11,15,24^. In a recent study, RTD-1 was highly effective in promoting fungal clearance in mice infected systemically with MDR *C. albicans* which resulted in markedly enhanced long-term survival^15^.

Building on insights into pharmacologic and biophysical properties of θ-defensins, a library of minimized theta defensins (i.e., MTDs) was synthesized and evaluated for in vitro and in vivo properties in a multidimensional screen, details of which will be reported elsewhere. MTD12813 emerged as a promising lead for further preclinical evaluation as a therapeutic for MDR bacterial infections. Here we report that MTD12813 is efficacious in treatment of CRE sepsis in BALB/c mice and acts by stimulating host-mediated clearance of CRE pathogens through enhancement in phagocytosis and immune cell recruitment, while concomitantly modulating pathogenic cytokine responses.

## Results

### In vitro antibacterial activity of MTD12813

Minimum inhibitory concentrations (MICs) of MTD12813 and RTD-1 against a panel of CRE bacteria were determined and compared with carbapenem antibiotics (meropenem and imipenem), colistin, and polymyxin B. Test organisms included *K. pneumoniae* and *E. coli* strains expressing carbapenemases (KPC-2, NDM-1), extended-spectrum β-lactamase (ESBL) resistance factors as well as antibiotic sensitive reference strains (see Methods). Under standard CLSI assay conditions, the MIC of MTD12813 was 3.13–6.25 µg/ml against CRE and non-CRE strains of *K. pneumoniae* and *E. coli.* MTD12813 was superior to RTD-1, and was as or more effective than meropenem and imipenem against each of the strains tested. As expected, colistin and polymyxin B were effective at low concentrations (< 0.78 µg/ml) against all seven organisms. Of note, inclusion of 50% heat-inactivated mouse serum completely inhibited the activities of MTD12813 and RTD-1 against ^KPC+^*Kp* BAA-1705 (*Kp*-1705) (MIC > 100 µg/ml), a finding consistent with a recent study of the antifungal activity of RTD-1^15^. Similar to RTD-1, MTD12813 likely binds non-specifically to plasma proteins, reducing the amount of free peptide available for a direct antimicrobial effect. In contrast, the antimicrobial activities of the conventional antibiotics tested were unaffected or slightly enhanced by addition of serum (Table 1).

**Table 1:** MICs of MTD12813, RTD-1 and antibacterial drugs (μg/ml).

Strain ID	*K. pneumoniae*								*E. coli*					
	BAA-1705		BAA-2146		700603		BAA-1706		BAA-2340		BAA-2471		ML35	
MDR factor	KPC-2		NDM-1		ESBL		none		KPC-2		NDM-1		none	
MTD12813	6.25	> *100*	6.25	> *100*	6.25	> *100*	3.13	> *100*	6.25	> *100*	3.13	*100*	3.13	> *100*
RTD-1	> 100	> *100*	100	> *100*	> 100	> *100*	> 100	> *100*	50	> *100*	25	> *100*	25	> *100*
Meropenem	50	*50*	100	> *100*	< 0.78	< *0.78*	1.56	< *0.78*	6.25	*6.25*	50	> *100*	< 0.78	< *0.78*
Imipenem	> 100	*12.5*	> 100	*25*	< 0.78	< *0.78*	3.13	< *0.78*	25	*6.25*	50	*25*	< 0.78	< *0.78*
Colistin	< 0.78	< *0.78*	< 0.78	< *0.78*	< 0.78	< *0.78*	< 0.78	< *0.78*	< 0.78	< *0.78*	< 0.78	< *0.78*	< 0.78	< *0.78*
Polymyxin B	< 0.78	< *0.78*	< 0.78	< *0.78*	< 0.78	< *0.78*	< 0.78	< *0.78*	< 0.78	< *0.78*	< 0.78	< *0.78*	< 0.78	< *0.78*

Italics columns are MICs in the presence of 50% heat inactivated mouse serum.

### MTD12813 promotes survival in MDR bacterial septicemia

**Figure 2:**
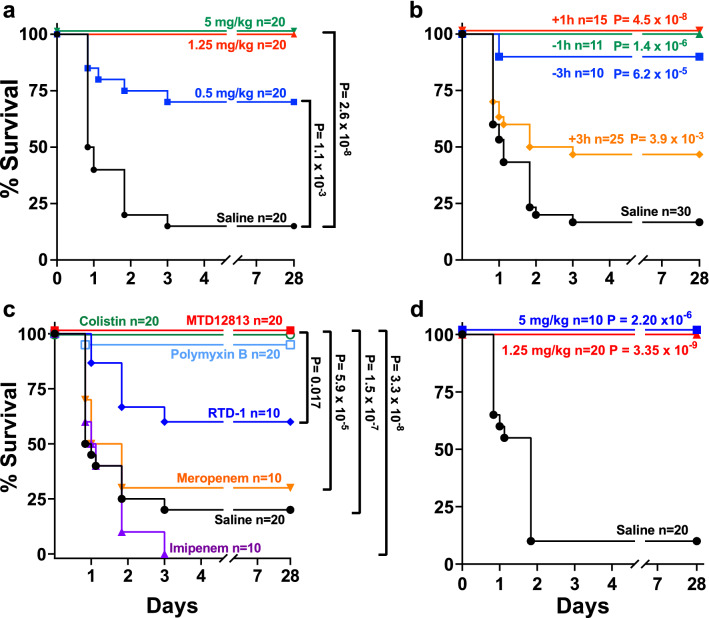
MTD12813 promotes survival in CRE bacteremic sepsis. (**a**-**c**) Mice were challenged i.p. with *Kp*-1705 (3 - 5 x 10^8^ CFU) at t = 0. (**a**) One h post infection (p.i.) mice were treated i.p. with MTD12813 at 0.5, 1.25, 5 mg/kg, or saline vehicle and monitored for up to 28 d. (**b**) Mice were treated with a single i.p. injection of 1.25 mg/kg MTD12813 before (-1 or -3 h) or after (+1 or +3 h) infection; controls received saline. P values compared to saline control. (**c**) Mice were treated 1 h p.i. with a single i.p. dose of 1.25 mg/kg MTD12813, meropenem, imipenem, colistin, or polymyxin B or 5 mg/kg RTD-1. (**d**) Mice were challenged i.p. with CRE *E. coli* BAA-2340 (3 - 6 x 10^7^ CFU). One h after infection, mice received a single i.p. injection of 5 or 1.25 mg/kg MTD12813 or saline. P values determined by Fisher’s exact test.

Adult BALB/c mice were infected intraperitoneally with *Kp*-1705, a virulent hypermucoid strain of *K. pneumoniae* (Methods) which results in rapid bacterial dissemination to blood, spleen, and other solid organs (liver, lungs, and kidneys; 10^6^–10^8^ CFU/g) within 1 h of infection (Supplemental Data Fig. 1). *Kp*-1705 infected mice showed clinical signs of systemic inflammatory response syndrome (SIRS)^25^, including diminished physical activity, hunched posture, piloerection, and altered breathing rates within 3 h of infection, and ~ 75% of vehicle control mice succumbed or were humanely euthanized by 96 h post infection (p.i.). Peptide (0.5, 1.25, or 5 mg/kg MTD12813) or saline treatment was initiated 1 h p.i.. A single dose of MTD12813 at 1.25 and 5 mg/kg dose levels resulted in 100% long term (≥ 28 days) survival (P = 2.6 × 10^–8^) (Fig. 2a). Compared to saline-treated controls, the clinical appearance/activity of mice treated with MTD12813 at 1.25 or 5 mg/kg markedly improved within 24 h of treatment, and behavior and appearance were normal by 96 h. Treatment efficacy, measured by survival, was reduced when the dose of MTD12813 was reduced to 0.5 mg/kg, but survival benefit was still statistically significant compared to saline treatment (P = 1.1 × 10^–3^).

The effect of timing of peptide treatment on *Kp*-1705 infected mice was analyzed in experiments in which a single dose of 1.25 mg/kg MTD12813 was administered 1 or 3 h before or after i.p. infection (Fig. 2b). All infected mice in the − 1 h and + 1 h treatment cohorts survived long term (P < 1 × 10^–6^), and nearly equivalent efficacy was obtained when mice were treated 3 h prior to infection. Even when treatment was initiated 3 h after infection, survival was enhanced (Fig. 2b; P = 3.9 × 10^–3^). Notably, all mice in the + 3 h cohort showed clinical signs of SIRS (described above) before MTD12813 treatment, consistent with the high tissue burdens of *Kp-*1705 in the blood and organs of infected mice (Supplemental Fig. 1). Moreover, as discussed below, plasma levels of proinflammatory cytokines were markedly elevated within 2–4 h of *Kp*-1705 infection in mice, consistent with severe sepsis. Nevertheless, half of the + 3 h cohort were rescued by a single dose of MTD12813 and were long term survivors.

### Comparison of MTD12813 and conventional antibiotics for efficacy in Kp-1705 septicemia

Equal single doses of MTD12813, imipenem, meropenem, colistin, and polymyxin B were administered i.p. 1 h after *Kp*-1705 challenge (as in Fig. 2a,b). MTD12813 and colistin treatments resulted in 100% survival, and polymyxin B was 95% effective, but neither carbapenem provided significant survival benefit compared to saline treated controls. Lack of imipenem and meropenem benefit was expected given that *Kp*-1705 is highly resistant to carbapenems. Note the superior efficacy of 1.25 mg/kg of MTD12813 compared to 5 mg/kg of natural θ-defensin RTD-1 in vivo (Fig. 2c).

### Efficacy of MTD12813 against MDR E. coli

After *Klebsiella* spp., *E. coli* is the second most common cause of CRE infections^1^. Given the efficacy of MTD12813 against *K. pneumoniae*, we hypothesized that this peptide would be efficacious in *E. coli* septicemia. Mice were challenged i.p. with ^KPC+^*E. coli* BAA-2340 and treated 1 h p.i. with a single injection of peptide at 1.25 or 5 mg/kg. As shown in Fig. 2d, 100% of mice in both MTD12813 treatment cohorts survived ≥ 28 days (*P* ≤ 2.2 × 10^–6^) compared to 10% survival in saline controls. As observed with MTD12813 treatment of *Kp*-1705 infected mice, peptide treated mice infected with *E. coli* BAA-2340 were healthier than saline controls 24 h p.i., and appearance and behavior were normal by 96 h.

### MTD12813 pharmacokinetics (PK)

As noted above, i.p. infection with *Kp*-1705 results in rapid dissemination into blood and tissues by 60 min (Supplemental Data Fig. 1). Since MTD12813 treatment initiated 1 or 3 h post infection is highly effective (Fig. 2b), it is evident that therapeutic efficacy occurs in the setting of widely disseminated infection. To understand the kinetics of peptide absorption and systemic distribution, single dose PK analysis was performed following administration of 1.25 mg/kg of MTD12813 in naïve male and female BALB/c mice. MTD12813 was rapidly absorbed from the peritoneum with T_max_ of 15 min post injection and a peak plasma concentrations (C_max_) of 0.251 ± 0.0798 µg/ml (Supplemental Fig. 2b). Consistent with these results, rapid peritoneal uptake of MTD12813 from the peritoneal fluid (PF) was measured, as the 125 µg/ml peptide infusate was reduced to ~ 7.5 µg/ml within 2 min of i.p. administration (Supplemental Fig. 2b). Of note, no bacterial killing was observed in vitro when *Kp*-1705 was incubated in PF containing 7.5 µg/ml peptide (Supplemental Fig. 2c), consistent with an indirect mode of action of MTD12813.

### MTD12813 promotes bacterial clearance, phagocytosis, and neutrophil recruitment in Kp-1705 septicemia

**Figure 3:**
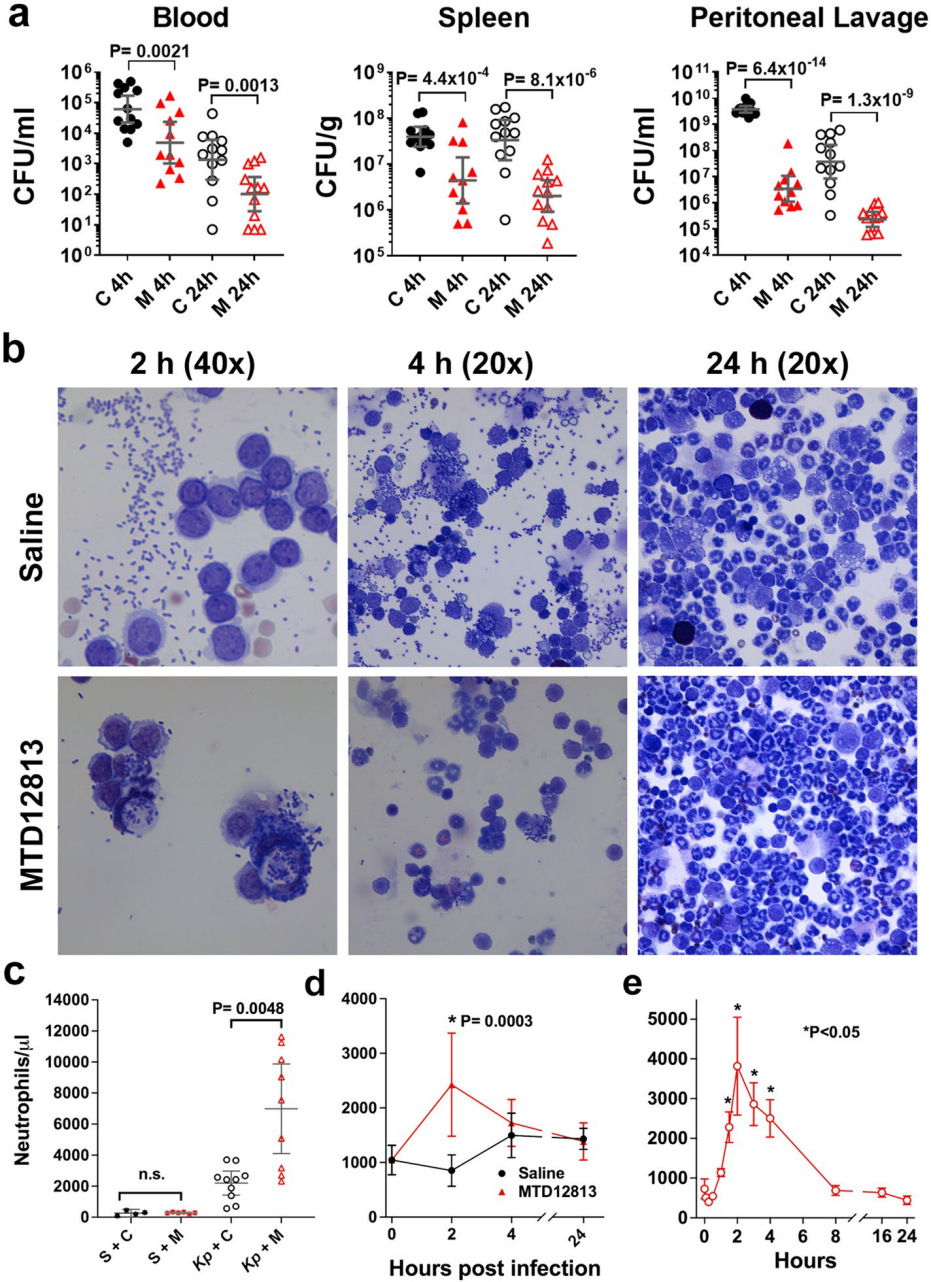
MTD12813 promotes bacterial clearance, neutrophil recruitment and phagocytosis in *Kp*-1705 bacteremia. (**a**-**d**) BALB/c mice were challenged i.p. with *Kp*-1705 (3 - 5 x 10^8^ CFU) and treated with 1.25 mg/kg MTD12813 (M) or saline control (C) 1 h p.i. (**a**) Mice were euthanized 4 or 24 h p.i. and bacterial burden determined. Data are geometric means ± 95% CI with P values calculated using Student’s t-test. (**b**) At 2, 4, and 24 h p.i., peritoneal lavage fluid was collected and cytospin preparations stained with H & E. (**c**) Peritoneal neutrophils were collected and counted 24 h p.i. from sham (S) and *Kp*-1705 (*Kp*) infected mice treated 1 h p.i. with saline (C) or 1.25 mg/kg MTD12813 (M). (**d**) Blood neutrophils from infected animals treated 1 h p.i. were quantified at t = 0, 2, 4 and 24 h, data shown as means ± 95%CI (n = 5 for sham n = 9-12 for trx groups), P values determined by Student’s t-test with Welch’s correction. (**e**) Naïve BALB/c mice (2M/2F per time point) received 1.25 mg/kg MTD12813 by a single i.p. injection, and blood neutrophil numbers were determined by CBC analysis. Graph depicts means ± SD, P values determined by Student’s t-test comparing each time point to t = 0.

Organs from long term survivors (28 days) of MTD12813-treated, *Kp*-1705 infected mice lacked culturable bacteria (data not shown), demonstrating that survival is associated with bacterial clearance. The effect of MTD12813 on bacterial clearance was evaluated in blood, spleen, and peritoneal lavage fluid (PLF) from mice 4 or 24 h after infection following a single dose treatment with saline or 1.25 mg/kg MTD12813 1 h p.i. (Fig. 3a). MTD12813 treatment significantly reduced bacterial burden in all three tissues at both 4 and 24 h (P < 0.003) (Fig. 3a). Within 3 h (4 h p.i.) of MTD12813 treatment, viable bacteria were reduced by 79.1%, 70.4%, and 99.5% in blood, spleen, and PLF, respectively, compared to saline treated animals. By 24 h, at which time MTD12813 treated mice showed marked improvements in clinical appearance and activity, bacterial burdens were further reduced by 93.8% in blood, 94.4% in spleen homogenates, and 99.8% in PLF, demonstrating that MTD12813 rapidly promotes bacterial clearance.

To further evaluate the effect of peptide treatment on bacterial clearance, cytospin preparations of PLF of *Kp*-1705 infected mice were evaluated 2, 4, or 24 h p.i. in MTD12813 and saline treated mice. Compared to saline controls, MTD12813-treated mice showed a marked increase in peritoneal cell associated or phagocytosed bacteria, and few extracellular bacteria were evident (Fig. 3b). Additionally, peptide treatment promoted a significant increase in peritoneal neutrophils 4 h p.i. (data not shown) which progressed to a threefold increase in PLF neutrophils at 24 h (P = 0.0048; Fig. 3c). The peptide-induced neutrophilic infiltrate was only observed in infected mice, as MTD12813 treatment of sham-infected mice showed no increase in neutrophil numbers 24 h after peptide delivery (Fig. 3c). In parallel, a transient peripheral blood neutrophilia occurred in infected mice treated with MTD12813 (but not saline) which peaked at ca. 2 h p.i. (Fig. 3d). By 4 h p.i., blood neutrophil counts in MTD12813-treated mice were not significantly different from those of mice treated with saline (Fig. 3d). Modest, but not statistically significant, elevations in monocytes at 2 h p.i. were observed in MTD12813 treated mice, while lymphocyte, eosinophil, and basophil levels showed no apparent differences among treatment groups (data not shown). Interestingly, a similar transient neutrophilia was observed in blood collected from naïve BALB/c mice treated with a single i.p. dose of MTD12813 (Fig. 3e).

### MTD12813 promotes bacterial phagocytosis

**Figure 4:**
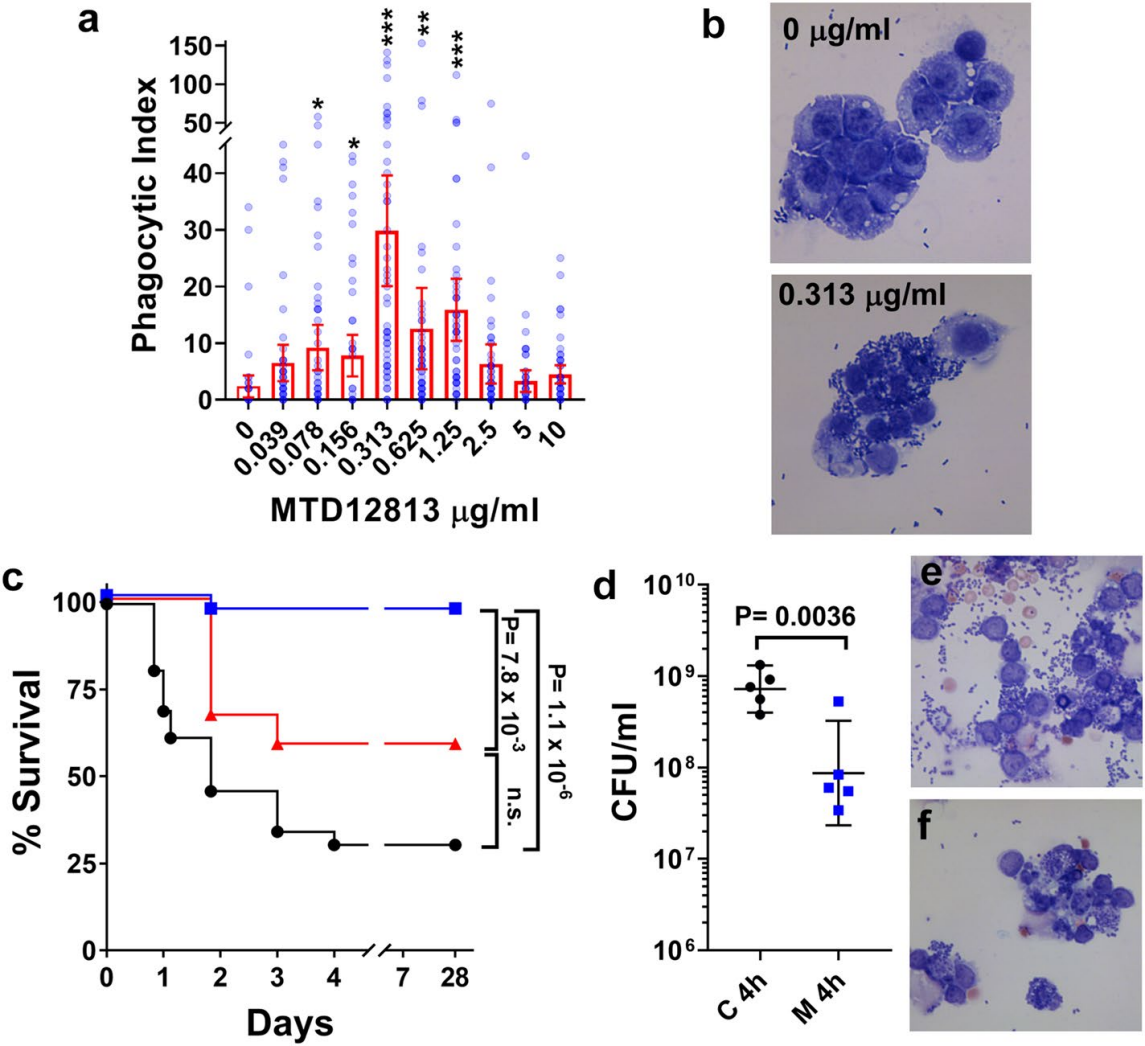
MTD12813 promotes phagocytosis of *Kp*-1705. (**a**-**b**) Phagocytosis by RAW 264.7 macrophages incubated with MTD12813 in the presence of *Kp*-1705 (50:1 MOI). (**a**) Cytospins were stained with H&E and phagocytic index (bacteria per cell) was determined manually. Scatter plots depict means ± 95% CI, P values determined by ANOVA with Uncorrected Fisher’s LSD post test, *P ≤ 0.05, **P < 0.01, ***P < 0.0001. (**b**) Images comparing effects of 0 and 0.313 µg/ml MTD12813 on phagocytic activity. (**c**) Mice were challenged i.p. with *Kp* BAA-1705 cells preincubated for 1 h prior to infection with saline (●, n = 26) or 1.25 µg/ml MTD12813 (■, n = 26). A control cohort received saline preincubated bacteria followed immediately by i.p. treatment with 500 µl of MTD12813 at 1.25 µg/ml (▲, n = 12). Survival of each cohort is shown. Experiment was repeated 4 times, P values determined by Fisher’s exact test. (**d**) PLF was collected from 5 mice challenged with saline (C) or MTD12813-pretreated *Kp*-1705 (as in panel c) that were euthanized 4 h p.i., and bacteria in each sample were quantified by plating (means ± SD; P values by Student’s t-test). H & E stained cytospin preparations from saline (**e**) and MTD12813-pretreated samples (**f**).

To further analyze the effect of MTD12813 on phagocytic activity, we analyzed the effect of the peptide on phagocytosis of *Kp*-1705 by mouse RAW 264.7 macrophages. Co-incubation of live *Kp*-1705 with RAW 264.7 cells with 0–10 µg/ml MTD12813 showed a concentration dependent inverted-U induction of phagocytosis (Fig. 4a). Maximal phagocytic activity was observed at 0.313 µg/ml, which resulted in a 12.6-fold increase in mean phagocytic index (P < 1 × 10^–6^_;_ Fig. 4a) relative to no peptide controls, and the increase was readily observed microscopically (Fig. 4b). Of note, maximal phagocytic activity was achieved at peptide levels approximating the plasma C_max_ values obtained following i.p. administration of 1.25 mg/kg MTD12813 (Suppl. Fig. 2).

The phagocytosis-inducing activity of MTD12813 was further analyzed by incubating live *Kp*-1705 cells with 1.25 µg/ml of MTD12813 (1/5 the MIC under optimal conditions; Table 1) and challenging mice with MTD12813 pre-treated bacteria. Although pre-treatment had no effect on bacterial viability or replication fitness of the organism (Supplemental Fig. 4), mice challenged with the bacteria-peptide mixture were protected (Fig. 4c). Because no statistically significant protection was afforded by i.p. administration of an equal quantity of peptide (0.5 ml of 1.25 µg/ml) immediately following infection, we inferred that preincubation of bacteria with MTD12813 renders them susceptible to host clearance. Also, pre-incubation with MTD12813 markedly increased bacterial killing and phagocytosis in the peritoneal cavity (Fig. 4d–f), consistent with the effect of peptide treatment on phagocytosis and bacterial killing of *Kp*-1705 infected mice (Fig. 3a,b).

### MTD12813 modulates cytokine responses in Kp-1705 induced sepsis

**Figure 5:**
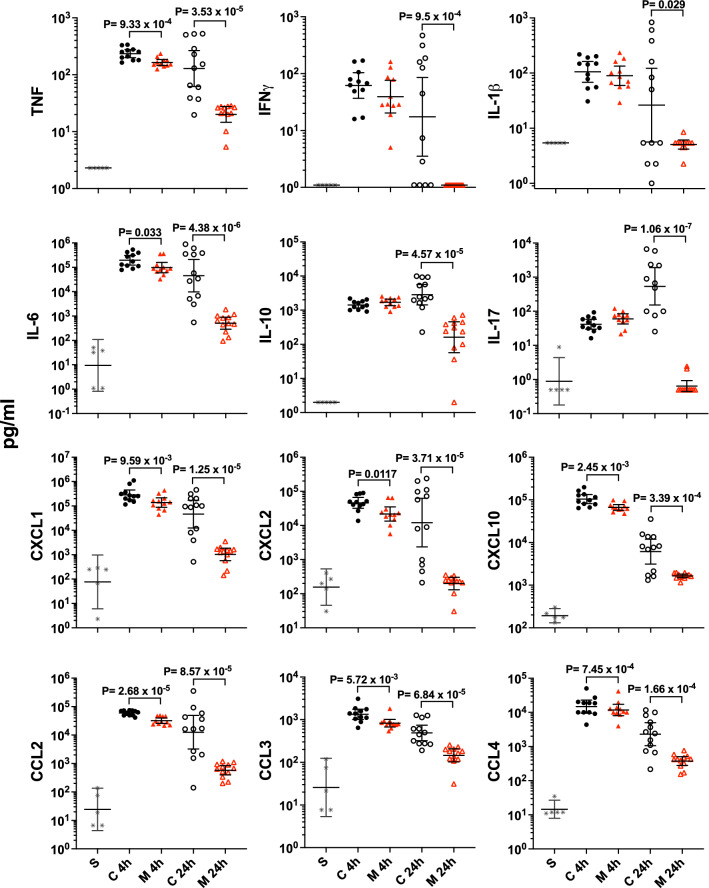
MTD12813 modulation of cytokine responses in *Kp*-1705 sepsis. Mice were challenged i.p. with *Kp*-1705 and treated 1 h later with MTD12813 (1.25 mg/kg i.p.) (M) or saline (C). The sham (S) cohort received bacteria-free suspension buffer at t = 0 followed by saline 1 h later. Mice were euthanized 4 or 24 h p.i. and plasma samples and cytokines quantified. P values were calculated using ANOVA with Uncorrected Fisher’s LSD.

We analyzed the effect of MTD12813 treatment on cytokines associated with host responses to infection that are implicated in the immunopathology of bacteremic sepsis. Multiplex analysis of 32 mouse cytokines was performed on plasma samples collected 2, 4, and 24 h p.i. from *Kp*-1705 septicemic mice treated with MTD12813 or saline (Fig. 5 and Supplemental Table 1). *Kp*-1705 infection induced a rapid and profound elevation of 26 cytokines, some of which increased > 1000 fold within 2–4 h p.i. (Supplemental Table 1). Plasma levels of six cytokines (IL-2, IL-3, IL-4, IL-7 and VEGF) were not significantly altered by *Kp*-1705 infection nor were they affected by MTD12813 treatment.

MTD12813 treatment had selective and differential effects on initial early (2 and 4 h p.i.) and later (24 h p.i.) cytokine responses in *Kp*-1705 infected mice (Fig. 5 and Supplemental Table 1). The earliest (2 h) effects seen were statistically significant reductions of CCL4 and CXCL2 (Supplemental Table 1). By 4 h p.i., 7 additional cytokines were significantly reduced, including TNF, IL-6, and CXCL1 (KC) which have major roles in sepsis pathogenesis (Fig. 5). MTD12813 treatment also resulted in significant increases of IL-9 and IL-15 levels at 4 h p.i. (Supplemental Table 1).

Although several early (2 or 4 h) cytokine responses were selectively regulated by MTD12813 treatment, there was a global reduction in all 26 plasma cytokines affected by *Kp*-1705 infection at the 24 h time point, including those that had been increased initially (IL-9 and IL-15; Fig. 5 and Supplemental Table 1). With the exception of IL-15 (P = 0.052), reductions of cytokine levels were significant compared to saline treated controls (Supplemental Table 1), and levels of 23 cytokines were reduced by > 80%, most notably TNF, (-89.8%; P = 3.52 × 10^–5^), IL-6 (− 99.7%; P = 4.38 × 10^–6^), CXCL1 (KC) (− 98.8%; P = 1.25 × 10^–5^), and IL-17 (− 100.0%; P = 1.06 × 10^–7^) (Fig. 5). Moreover, MTD12813 treatment restored 14 cytokines to sham control levels by 24 h (Fig. 5, Supplemental Table 1).

The effect of MTD12813 administration on plasma cytokines was also analyzed in uninfected mice that received saline (sham infection controls) followed 1 h later with a single i.p. dose of 1.25 mg/kg peptide. At the 4 h collection point, only three of the 32 mouse cytokines changed significantly relative to sham/saline controls: IL-5, IL-6, and CXCL1 were significantly elevated compared to vehicle treated controls (Supplemental Fig. 3). However, all three cytokines returned to baseline by 24 h.

### MTD12813 modulates cytokine responses in vitro

**Figure 6:**
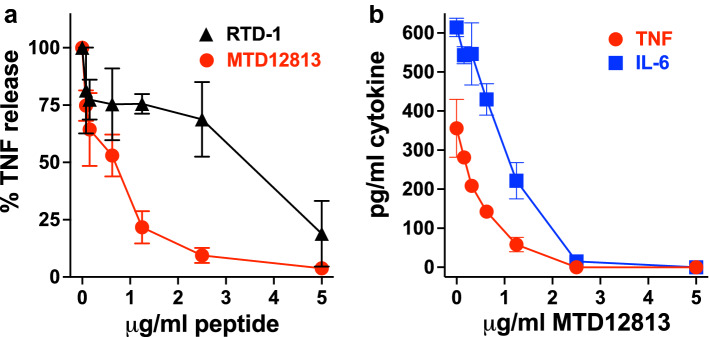
In vitro MTD12813 modulation of cytokine responses. (**a**) LPS-stimulated RAW 264.7 cells were incubated with MTD12813 (●) or RTD-1 (▲) for 2 h and supernatant TNF quantified by ELISA. (**b**) Human whole blood was incubated for 4h with 1000 CFU/ml of *Kp*-1705 with MTD12813, and supernatant TNF (●) and IL-6 (■) were quantified by ELISA.

The effects of MTD12813 and RTD-1 on cytokine responses was further analyzed by incubating LPS-stimulated mouse RAW 264.7 macrophages with 0–5 µg/ml MTD12813 or RTD-1. Consistent with previous studies^26^, RTD-1 suppressed the release of soluble TNF from LPS stimulated macrophages dose dependently (Fig. 6a). MTD12813 also inhibited LPS-stimulated TNF release, but 50% TNF suppression was obtained at ca. fourfold lower concentration than RTD-1. To address the possibility that TNF suppression by MTD12813 was the result of LPS neutralization by the peptide, we evaluated the effect of MTD12813 on LPS activity using the limulus amebocyte lysate assay as described previously^11^ (see “Methods”). No LPS inhibition by the peptide was detected at any concentration used in the RAW cell assays (data not shown), indicating the MTD12813 modulates the macrophage inflammatory response to endotoxin. Of note, the LPS concentration used in the RAW cell assays was 50-fold higher than that used to analyze potential inhibition of LPS by MTD12813.

We then tested the effect of MTD12813 on cytokines released by human whole blood incubated with live *Kp*-1705 cells. As shown in Fig. 6b, MTD12813 concentration-dependently suppressed the release of TNF and IL-6. Peptide alone had no effect on TNF or IL-6 release (data not shown). These results indicate that the effects of MTD12813 in modulating cytokine responses in vivo in mice and in mouse RAW 264.7 macrophages extend to TNF and IL-6 release by human blood leukocytes.

### MTD12813 stability

Preliminary studies of MTD12813 indicated that the peptide is exceedingly stable in aqueous media including acidic solutions as low as pH 2.0 (data not shown). To assess peptide stability further, MTD12813 was incubated in either human EDTA-plasma or serum (90% vol/vol) at 37 °C for 48 h. Reversed-phase UPLC analyses showed that > 95% of the peptide remained intact following incubation in both matrices. In addition, 1.25 µg/ml MTD12813 was incubated with log-phase *Kp*-1705 or *E. coli* ATCC-BAA-2340 suspended in 50 mM HEPES at 37 °C for 24 h. Intact MTD12813 was quantified by LC–MS/MS which showed 72.4 ± 0.93% and 81.3 ± 2.1% of MTD12813 was found intact post *Kp*-1705 and *E. coli* incubations respectively. These findings demonstrate that MTD12813 is remarkably resistant to bacterial proteases.

## Discussion

MTD12813 is a bioinspired macrocyclic peptide that is structurally related to naturally occurring θ-defensins. RTD-1, the prototype θ-defensin, was identified as a broad-spectrum antimicrobial peptide in vitro^8,9^. Subsequent studies demonstrated that RTD-1 homeostatically regulates inflammation in infectious and non-infectious preclinical models^11,12,15,17,18,27^. Mechanisms implicated in the moderation of cytokine-driven inflammation include modulation of NFκB and MAPK pathways^13^, suppression of TNF release via the inhibition of the pro-TNF sheddase TACE (ADAM17)^26^, and disease resolution and normalization of gene signatures in models of rheumatoid arthritis^18,27^. Also, systemic administration of RTD-1 promoted fungal clearance and long-term survival in mouse models of disseminated candidiasis^15^. RTD-1 efficacy in this model was not the result of a direct antifungal effect of the peptide, since the plasma C_max_ value was < 1% of the MIC of the peptide^15^. RTD-1 treatment was superior to treatment with fluconazole, and antifungal efficacy correlated with transient induction of peripheral blood neutrophilia and normalization of proinflammatory cytokines including TNF, IL-1β, IL-17, and IL-6^15^.

Among a series of minimized θ-defensins, MTD12813 was identified as a promising candidate based on preliminary studies demonstrating its low toxicity in vitro and in vivo, stability in biological matrices, and suppression of TNF release by LPS-stimulated THP-1 macrophages. Here we show that MTD12813 is highly effective in promoting long term survival, bacterial clearance, and moderation of dysregulated systemic inflammation in CRE septicemia.

Both therapeutic and prophylactic single dose administration of MTD12813 significantly enhanced long term survival following challenge with *Kp*-1705, an organism that is resistant to carbapenems in vitro and in vivo*.* Moreover, the peptide was as effective in vivo as colistin and polymyxin B, drugs of last resort that have limited utility as human therapeutics owing to their toxicities^28^. RTD-1 was also effective in *Kp*-1705 sepsis, but at approximately tenfold higher concentrations than the effective dose of MTD12813. In addition, MTD12813 promoted long term survival in mice challenged with a CRE strain of *E. coli* (BAA-2340; Fig. 2d) demonstrating efficacy against the two most common species of CRE pathogens.

Highly efficacious single dose treatment of both CRE pathogens was obtained with administration of MTD12813 at 1.25 mg/kg, a dose level that resulted in plasma C_max_ of 0.25 µg/ml which is > 400 times lower than the MIC of MTD12813 against both organisms in the presence of heat-inactivated serum (> 100 µg/ml). Moreover, MTD12813-containing peritoneal fluid had no bactericidal activity against *Kp*-1705. This demonstrates that the antimicrobial effect of MTD12813 is not direct, but rather is mediated by stimulation of host clearance mechanisms, similar to the therapeutic effect of RTD-1 in systemic candidiasis^15^.

In the bacteremia model employed, *Kp*-1705 was widely disseminated in blood and solid organs at the time of MTD12813 treatment. This, and the lack of MTD12813 activity in peritoneal fluid, enabled infection and delayed peptide administration by the same route. Peptide treatment induced marked reductions in bacterial burden in blood, peritoneal lavage, and spleen within 4 h of treatment, and this was accompanied by a marked peptide-induced neutrophilic infiltrate in the peritoneal cavity. Microscopic analysis of PLF from MTD12813 treated infected mice revealed markedly increased phagocytosis of bacteria compared to control. Blood neutrophil levels were also transiently elevated in infected mice treated with MTD12813 as well as in mock-infected controls, suggesting that the peptide stimulates a systemic neutrophilic response. Of note, i.p. administration of MTD12813 induced transient rises in CXCL1, IL-5, and IL-6 in sham treated mice. CXCL1 is a potent neutrophil chemokine which in concert with its receptor CXCR2, plays a key role as one of two cytokine/receptor axes controlling the migration of neutrophils from the bone marrow^29^. Thus, CXCL1 may mediate MTD12813-induced transient neutrophilia.

MTD12813-mediated phagocytosis, revealed by cytologic examination of PLF in infected mice, was recapitulated in vitro by co-incubation of *Kp*-1705 with RAW 264.7 macrophages in the presence of nanomolar concentrations of the peptide, resulting in up to 12-fold increases in phagocytic index. Consistent with this, preincubation of *Kp*-1705 with a sub-MIC concentration of MTD12813 promoted phagocytosis in vivo and markedly enhanced mouse survival (Fig. 4c), evidence that MTD12813 has opsonin-like properties. Mice were also protected from *Kp*-1705 septicemia by a single dose of MTD12813 administered 1 or 3 h prior to infection, indicating that systemic levels of the peptide are also effective prophylactically. The degree to which this effect is mediated by induction of phagocytosis in extraperitoneal tissues remains to be determined.

Pathogenesis of septicemia results from an imbalance of the host damage-response framework^30^. Challenge with *K. pneumoniae* initiates host innate immune responses which progress through systemic inflammatory response syndrome, evolving to severe sepsis, and finally in lethal septic shock. This detrimental host response is largely driven by dysregulated cytokine signaling which results in uncontrolled inflammation, host-mediated tissue destruction, disseminated intravascular coagulation, and multiple organ failure^25,31,32^. Modulation of cytokine responses in MTD12813 treated mice was associated with suppression of otherwise immunopathologic cytokine release without inducing an immunocompromised state. Moreover, MTD12813 treatment significantly enhanced the recruitment of neutrophils to the peritoneal cavity, an innate immune response which is often accompanied by inflammatory tissue damage. However, MTD12813-induced neutrophil recruitment was accompanied by increased phagocytosis, bacterial clearance, and homeostatic resolution of plasma cytokines.

Although mouse models of bacteremic sepsis are distinct from human sepsis syndromes, they are a common starting point for identification of infectious disease drug candidates^33–36^. To address the potential of MTD12813 as a human therapeutic, we analyzed the immunomodulatory activity of the peptide on TNF and IL-6 expression by bacteria-stimulated human blood leukocytes. The peptide suppressed release of both cytokines in a concentration dependent manner. Other experiments showed that MTD12813 is remarkably stable in plasma and when incubated for 24 h with live CRE isolates, suggesting that this macrocyclic peptide is an attractive candidate for further preclinical development. Additional studies showed that i.p. administration of MTD12813 is well tolerated in BALB/c mice at doses more than 100-fold higher than the 0.5 mg/kg efficacious dose (data not shown).

In recent years there has been a dramatic increase in the development and approval of peptide drugs^37,38^. However, this has not been the case for clinical development of direct acting antimicrobial peptides (AMPs)^39,40^. Historic challenges in clinical development of AMP-based drugs have been due to toxicity, poor activity in biological matrices, or poor bioavailability/pharmacokinetics^40^. More recent efforts have focused on the development of anti-infective peptides that have host directed modes of action. Examples include innate defense regulator (IDR) -1018 and CD28 mimetic AB103 (Reltecimod™). IDR-1018 is an immunomodulatory dodecapeptide amide that was bioinspired by the bovine cathelicidin Bac2a. IDR-1018 was effective in treatment of systemic candidiasis in mice, with efficacy mediated by modulation of proinflammatory cytokines^41^. AB103 (Reltecimod™) is a CD28 mimetic that disrupts T-cell CD28 stimulatory pathways by blocking the binding of exotoxin superantigens to CD28 and preventing downstream overactivation of T-cell driven proinflammatory cytokine responses^42,43^. The potential of immunomodulatory peptides was illustrated after a Phase III human trial of AB103 was completed for treatment of necrotizing soft-tissue infections^44,45^.

Here, we report the first use of a θ-defensin-inspired macrocyclic peptide for treatment of Gram negative bacterial infections. Like RTD-1, the mechanism of action of MTD12813 is host directed, and therefore, less likely to select for drug resistance than conventional antibiotics^46^. Moreover, the stability and pharmacologic properties of MTD12813 suggests that it represents a promising new class of immunomodulating anti-infectives much needed to address the worldwide crisis resulting from antimicrobial resistance.

## Materials and methods

### Ethics

All methods were performed in accordance with relevant federal, state, and institutional guidelines. All animal studies were performed in compliance with ARRIVE guidelines 2.0. Animal use protocols were approved by The University of Southern California (USC) Institutional Animal Care and Use Committee (IACUC), Protocol #20538. Blood was obtained from healthy adult volunteers according to approved USC Institutional Review Board (IRB) Protocol HS-09-00280.

### Peptides and antibiotics

The hydrochloride salts of MTD12813 and RTD-1 (> 95%) were produced by solid phase peptide synthesis as described^10,14^. Imipenem (I0160), meropenem (M2574), ceftazidime (A6987), colistin (C4461), and polymyxin B (P0972) were purchased from Sigma-Aldrich (St. Louis, MO). Stock solutions of MTD12813, RTD-1, and antibacterial drugs were prepared as concentrated stocks in sterile water. For animal injection, peptides and antibacterial drugs were diluted to the indicated concentrations in sterile normal saline.

### Bacterial strains

Bacterial strains were obtained from American Type Culture Collection. *K. pneumoniae* strains included BAA-1705 (*bla*_KPC-2_, hypermucoid), BAA-2146 (*bla*_NDM-1_), BAA-700603 (SHV-18, extended spectrum β-lactamase, ESBL), and BAA-1706 (non-CRE reference strain). *E. coli* strains included BAA-2340 (*bla*_KPC-2_), BAA-2471 (*bla*_NDM-1_), and ML35 (non-CRE reference strain).

### Antibacterial assays

Minimum inhibitory concentration (MIC) assays were performed ± 50% heat-inactivated mouse serum using Clinical and Laboratory Standards Institute (CLSI; document M07-A9) protocols. Bacteria (5 × 10^5^ CFU/ml) were incubated with antibiotics or peptide for 20 h at 37 °C, and bacterial growth was determined by A_620_ using a SpectraMax M5e plate reader. MIC was the lowest agent concentration that completely inhibited growth as determined by A_620_ absorbance.

### *K. pneumoniae* and *E. coli* bacteremia

BALB/c mice (Jackson Laboratories) 8–10 weeks of age were acclimated (3–5 to a cage for at least 5 days prior to infection) in a thermostatically controlled room with 12 h light/dark cycle. Cryopreserved *K. pneumoniae *^47^ cells were thawed and adjusted to a density of 6–10 × 10^8^ CFU/ml, confirmed by plating on trypticase soy agar (TSA) plates. *E. coli* BAA-2340 was grown to log-phase in trypticase soy broth, pelleted, washed with PBS, and resuspended in PBS at 0.5–1 × 10^9^ CFU/ml. Mice were infected by i.p. injection of 2–5 × 10^8^ CFU of *Kp*-1705 or 3–6 × 10^7^ CFU of *E. coli* BAA-2340 using a 28 g needle in a volume of 0.5 ml. Post-infection, mice were observed three times a day for at least 4 days and then daily for 28 days. Mice were euthanized when they became moribund. All experiments used both male and female mice and were repeated at least twice.

### Bacterial burden determination

Blood, peritoneal lavage fluid, and spleen homogenates were obtained from mice following by CO_2_ euthanasia. Citrate-anti-coagulated blood was collected by aseptic cardiac puncture. Peritoneal lavage was performed by injecting 3 ml of PBS into the peritoneal cavity, massaging the abdomen, and then collecting fluid aseptically through a minor incision in the abdominal wall. Spleen was surgically removed and homogenized in sterile PBS using Polytron PT10-35 homogenizer. Samples were serially diluted and plated in triplicate on TSA plates, incubated overnight at 37 °C and colonies counted.

### Hematology and cytokine analyses

EDTA-anticoagulated blood was collected aseptically by terminal cardiac puncture and analyzed for complete blood cell count using an Element HT5 hematology analyzer (Heska). Blood plasma and clarified peritoneal lavage were prepared by two-step centrifugation described previously^24^. Plasma cytokine levels were quantified using a mouse-specific MILLIPLEX MAP kit (Millipore Cat# MCYTMAG-70K) as described^11^.

### MTD12813 pharmacokinetics

Single dose PK of MTD12813 was evaluated by quantifying plasma peptide concentrations following 1.25 mg/kg injections administered i.p. to 2 M/2F BALB/c mice. EDTA plasma was prepared from blood collected aseptically by terminal cardiac puncture. MTD12813 plasma concentrations were determined by reverse-phase liquid chromatography (XBridge phenyl 3.5 µm column, Waters) on an Acquity H-Class UPLC (Waters) with tandem electrospray mass spectroscopy on a Xevo TQ-S running MassLynx V4.1 (Waters). Quantitative mass spectroscopy was performed by multiple-reaction monitoring transition 394.04 > 519.35, with area under the curve determined by TargetLynx (Waters).

### Cell culture

Mouse RAW 264.7 macrophages (RAW cells; ATCC, TIB-71) were grown in Dulbecco’s Modified Eagle Medium (DMEM) with 10% heat-inactivated fetal bovine serum (HI-FBS) with 100 U/ml penicillin/streptomycin, and suspended to 2–3 × 10^6^ cells/ml in fresh media before use.

### Phagocytosis assay

*Kp*-1705 was grown overnight, harvested by centrifugation, washed twice, resuspended in Hank’s Balanced Salt Solution (HBSS), and adjusted to a density of 2.5 × 10^8^ CFU/ml. Bacteria were pre-incubated with varied concentrations of MTD12813 at room temperature for 30 min, and incubated (MOI 50:1) with RAW cells (2.5 × 10^6^ cell/ml) with orbital shaking at 37 °C for 30 min. Cytospin preparations of 100 µl (2.5 × 10^5^ RAW cells/slide) samples were then prepared, fixed with methanol, and stained with hematoxylin and eosin (H & E). Slides were viewed at 100× and 50 RAW cells selected from random fields were manually counted for number of cell associated bacteria. All samples were run in duplicate and experiments performed twice. RAW cell viability post incubation was determined in parallel using trypan blue stain and SYTOX Green nucleic acid viability stain, both of which demonstrated cell viability of > 95%.

### LPS stimulation of RAW cells

RAW cells were seeded into 48-well tissue culture plates at 1–1.5 × 10^5^ cells/well and allowed to adhere overnight. Cells were washed twice and suspended in DMEM with 1% FBS with 0–5 µg/ml of MTD12813 immediately followed by addition of 5 ng/ml (25 EU/ml) of *E. coli* 0111:B4 LPS (Sigma L4391). Plates were incubated for 2 h at 37 °C in 5% CO_2._ Well contents were removed, clarified by centrifugation, and supernatants frozen at − 80 °C. Supernatant TNF was quantified by mouse TNF ELISA (Invitrogen, Cat #BMS607-3).

### LPS neutralization analysis

A limulus amebocyte lysate endotoxin detection assay (Pierce A39553) protocol was employed by preincubating 0.5 EU/ml of LPS with either MTD12813, RTD-1, or colistin (0.078–5 μg/ml) in H_2_0 at 37 °C for 30 min followed by quantifying free LPS per manufacturer’s instructions. All samples were compared to a LPS standard curve to determine the degree of LPS neutralization.

### Bacterial stimulation of human blood

Log-phase *Kp-*1750 grown in TSB were pelleted, washed twice in PBS, and suspended to 1 × 10^5^ CFU/ml in RPMI-1640. EDTA-anticoagulated blood collected from a healthy adult human donor was incubated at a final dilution of 1:10 in RPMI-1640 with 1000 CFU/ml of *Kp-*1705 and varied concentrations of MTD12813 for 4 h 37 °C in 5% CO_2_ with gentle rocking. Incubation supernatants were frozen at − 80 °C. TNF and IL-6 were quantified by ELISA (Invitrogen KHC3011, KHC0061).

### Peptide stability analysis

MTD12813 (final concentration of 100 µg/ml) was added to either 90% human EDTA plasma or 90% human serum and incubated at 37 °C for 48 h. Samples were processed by addition of 10% HOAc and 5% ACN (final concentrations) at time 0 and 48 h, and MTD12813 was quantified by C_18_ RP-HLPC on an Acquity H-Class UPLC with an analytical PDA detector using Empower 3 software (Waters). All samples were performed with technical replicates and analyzed by UPLC in duplicate and the experiment repeated once. In separate experiments, MTD12813 (1.25 µg/ml final) was incubated with log phase *Kp-*1705 (5 × 10^7^ CFU/ml) in 50 mM HEPES pH 7.4. A time 0 h sample was collected, and the remaining suspension incubated at 37 °C for 24 h. Samples were processed by addition of 5% formic acid/5% acetonitrile, vortexed, and clarified at 22,000×*g* for 10 min. Supernatant MTD12813 was then quantified by LC–MS/MS as described in PK section above. All samples were performed with technical replicates and analyzed by LC–MS/MS in duplicate.

### In vivo phagocytosis and peritoneal bioburden determination

Cryopreserved *Kp-*1705 was suspended in PBS to a density of ca. 1 × 10^9^ CFU/ml, and incubated with saline or 1.25 µg/ml MTD12813 at room temperature for 1 h. Mice (n = 26) were then infected i.p. with 500 µl of saline- or MTD12813- pretreated *Kp-*1705, and a third cohort (n = 12) received 500 µl i.p. of 1.25 µg/ml MTD12813 immediately following infection with saline treated *Kp*-1705. Five mice from each cohort were euthanized 4 h p.i., and peritoneal lavage was collected for bacterial burden determination and cytospin preparation. In vitro antibacterial activity of MTD12813 (1.25 μg/ml) -treated cells (1 h; 37 °C) was determined by plating saline or peptide treated cells on TSA plates. Growth kinetics of saline and MTD12813-treated cells was determined by diluting samples from the 1 h incubation mixtures 1:40 into TSB and monitoring growth (620 nm) for 5.5 h at 37 °C. Antibacterial activity of MTD12813 in PF was determined by i.p. injection of naïve male BALB/c mice (n = 5 per group) with 1.25 mg/kg of MTD12813 or saline i.p. (10 ml/kg). Mice were euthanized 1 h after i.p. injection and peritoneal fluid was harvested aseptically using a micropipette introduced into a surgical incision in the abdominal wall. Peritoneal fluid was clarified by centrifugation at 1200×*g* and an aliquot used to quantify MTD12813 by LC–MS/MS, as described above. Antimicrobial activity was determined by incubating PF (9:1 vol/vol) with *Kp*-1705 at a final density of 3 × 10^7^ CFU/ml. Samples were incubated at 37 °C for 1 h, serially diluted, and plated on TSB to determine bacterial viability.

### Statistical analyses

All statistical analyses were performed using GraphPad Prism Version 8.2.1. End-point survival data were compared using Fisher’s exact test. P values of ≤ 0.05 were defined as significant. Statistics analyzing bacterial burdens and cytokine responses first confirmed lognormal distribution by Anderson–Darling test. Values were then log-transformed and analyzed by one-way ANOVA with an Uncorrected Fisher’s Least Significant Difference (LSD) post-test. Student’s t-tests were performed using a two-tail analysis. All other statistical analyses are described in figure legends.

## Supplementary Information

Supplementary Information.

## Data Availability

The datasets generated during and/or analyzed during the current study are available from the 350 corresponding author on reasonable request.

## References

[1] Centers for Disease Control and Prevention. Antibiotic Resistance Threats in the United States. (US Department of Health and Human Services CDC, 2013).

[2] Oneill J (2016). Tackling Drug-Resistant Infections Globally: Final Report and Recommendations.

[3] Papp-Wallace KM, Endimiani A, Taracila MA, Bonomo RA (2011). Carbapenems: Past, present, and future. Antimicrob. Agents Chemother..

[4] Winkler ML (2015). Unexpected challenges in treating multidrug-resistant Gram-negative bacteria: Resistance to ceftazidime-avibactam in archived isolates of Pseudomonas aeruginosa. Antimicrob. Agents Chemother..

[5] Poirel L, Pitout JD, Nordmann P (2007). Carbapenemases: Molecular diversity and clinical consequences. Future Microbiol..

[6] Pitout JD, Nordmann P, Poirel L (2015). Carbapenemase-producing klebsiella pneumoniae, a key pathogen set for global nosocomial dominance. Antimicrob. Agents Chemother..

[7] Munoz-Price LS (2013). Clinical epidemiology of the global expansion of *Klebsiella pneumoniae* carbapenemases. Lancet Infect. Dis..

[8] Tran D (2002). Homodimeric theta-defensins from rhesus macaque leukocytes: Isolation, synthesis, antimicrobial activities, and bacterial binding properties of the cyclic peptides. J. Biol. Chem..

[9] Tongaonkar P (2011). Rhesus macaque {theta}-defensin isoforms: Expression, antimicrobial activities, and demonstration of a prominent role in neutrophil granule microbicidal activities. J. Leukoc. Biol..

[10] Garcia AE, Osapay G, Tran PA, Yuan J, Selsted ME (2008). Isolation, synthesis, and antimicrobial activities of naturally occurring theta-defensin isoforms from baboon leukocytes. Infect. Immunol..

[11] Schaal JB (2012). Rhesus macaque theta defensins suppress inflammatory cytokines and enhance survival in mouse models of bacteremic sepsis. PLoS ONE.

[12] Bensman TJ (2017). Efficacy of rhesus theta-defensin-1 in experimental models of pseudomonas aeruginosa lung infection and inflammation. Antimicrob. Agents Chemother..

[13] Tongaonkar P (2015). Rhesus macaque theta-defensin RTD-1 inhibits proinflammatory cytokine secretion and gene expression by inhibiting the activation of NF-kappaB and MAPK pathways. J. Leukoc. Biol..

[14] Tang YQ (1999). A cyclic antimicrobial peptide produced in primate leukocytes by the ligation of two truncated alpha-defensins. Science.

[15] Basso V (2019). Rhesus theta defensin 1 promotes long term survival in systemic candidiasis by host directed mechanisms. Sci. Rep..

[16] Wohlford-Lenane CL (2009). Rhesus theta-defensin prevents death in a mouse model of severe acute respiratory syndrome coronavirus pulmonary disease. J. Virol..

[17] Jayne JG (2018). Rhesus theta-defensin-1 attenuates endotoxin-induced acute lung injury by inhibiting proinflammatory cytokines and neutrophil recruitment. Am. J. Respir. Cell Mol. Biol..

[18] Schaal JB (2017). Suppression and resolution of autoimmune arthritis by rhesus theta-defensin-1, an immunomodulatory macrocyclic peptide. PLoS ONE.

[19] Lehrer RI, Lu W (2012). Alpha-defensins in human innate immunity. Immunol. Rev..

[20] Haudek SB (2003). Lipopolysaccharide dose response in baboons. Shock.

[21] Reichgott MJ, Melmon KL, Forsyth RP, Greineder D (1973). Cardiovascular and metabolic effects of whole or fractionated gram-negative bacterial endotoxin in the unanesthetized Rhesus monkey. Circ. Res..

[22] Wessels BC (1988). Plasma endotoxin concentration in healthy primates and during *E. coli*-induced shock. Crit. Care Med..

[23] Lehrer RI, Cole AM, Selsted ME (2012). theta-Defensins: Cyclic peptides with endless potential. J. Biol. Chem..

[24] Schaal JB (2018). Macrocyclic theta-defensins suppress tumor necrosis factor-alpha (TNF-alpha) shedding by inhibition of TNF-alpha-converting enzyme. J. Biol. Chem..

[25] Shrum B (2014). A robust scoring system to evaluate sepsis severity in an animal model. BMC Res. Notes.

[26] Schaal JB (2018). Macrocyclic θ-defensins suppress tumor necrosis factor-α (TNF-α) shedding by inhibition of TNF-α-converting enzyme. J. Biol. Chem..

[27] Tongaonkar P (2019). RTD-1 therapeutically normalizes synovial gene signatures in rat autoimmune arthritis and suppresses proinflammatory mediators in RA synovial fibroblasts. Physiol. Genom..

[28] Tsuji BT (2019). International Consensus Guidelines for the Optimal Use of the Polymyxins: Endorsed by the American College of Clinical Pharmacy (ACCP), European Society of Clinical Microbiology and Infectious Diseases (ESCMID), Infectious Diseases Society of America (IDSA), International Society for Anti-infective Pharmacology (ISAP), Society of Critical Care Medicine (SCCM), and Society of Infectious Diseases Pharmacists (SIDP). Pharmacotherapy.

[29] Lerman YV, Kim M (2015). Neutrophil migration under normal and sepsis conditions. Cardiovasc. Hematol. Disord. Drug Targets.

[30] Casadevall A, Pirofski LA (2003). The damage-response framework of microbial pathogenesis. Nat. Rev. Microbiol..

[31] Buras JA, Holzmann B, Sitkovsky M (2005). Animal models of sepsis: Setting the stage. Nat. Rev. Drug Discov..

[32] Doi K, Leelahavanichkul A, Yuen PS, Star RA (2009). Animal models of sepsis and sepsis-induced kidney injury. J. Clin. Invest..

[33] Murando F, Peloso A, Cobianchi L (2019). Experimental abdominal sepsis: Sticking to an awkward but still useful translational model. Mediators Inflamm..

[34] Zhao M, Lepak AJ, Andes DR (2016). Animal models in the pharmacokinetic/pharmacodynamic evaluation of antimicrobial agents. Bioorg. Med. Chem..

[35] Lewis AJ, Seymour CW, Rosengart MR (2016). Current murine models of sepsis. Surg. Infect. (Larchmt.).

[36] Poli-de-Figueiredo LF, Garrido AG, Nakagawa N, Sannomiya P (2008). Experimental models of sepsis and their clinical relevance. Shock.

[37] Lau JL, Dunn MK (2018). Therapeutic peptides: Historical perspectives, current development trends, and future directions. Bioorg. Med. Chem..

[38] Lee AC, Harris JL, Khanna KK, Hong JH (2019). A comprehensive review on current advances in peptide drug development and design. Int. J. Mol. Sci..

[39] Chen CH, Lu TK (2020). Development and challenges of antimicrobial peptides for therapeutic applications. Antibiotics (Basel)..

[40] Mookherjee N, Anderson MA, Haagsman HP, Davidson DJ (2020). Antimicrobial host defence peptides: Functions and clinical potential. Nat. Rev. Drug Discov..

[41] Freitas CG (2017). An immunomodulatory peptide confers protection in an experimental Candidemia Murine model. Antimicrob. Agents Chemother..

[42] Ramachandran G (2015). CD28 homodimer interface mimetic peptide acts as a preventive and therapeutic agent in models of severe bacterial sepsis and gram-negative bacterial peritonitis. J. Infect. Dis..

[43] Ramachandran G (2013). A peptide antagonist of CD28 signaling attenuates toxic shock and necrotizing soft-tissue infection induced by *Streptococcus pyogenes*. J. Infect. Dis..

[44] Bulger EM (2017). Validation of a clinical trial composite endpoint for patients with necrotizing soft tissue infections. J. Trauma Acute Care Surg..

[45] Bulger EM (2014). A novel drug for treatment of necrotizing soft-tissue infections: A randomized clinical trial. JAMA Surg..

[46] Chiang CY (2018). Mitigating the impact of antibacterial drug resistance through host-directed therapies: Current progress, outlook, and challenges. MBio.

[47] Nielsen TB, Bruhn KW, Pantapalangkoor P, Junus JL, Spellberg B (2015). Cryopreservation of virulent *Acinetobacter baumannii* to reduce variability of in vivo studies. BMC Microbiol..

