# Supplementary material for: A host-directed macrocyclic peptide therapeutic for MDR gram negative bacterial infections
Source: Sci Rep. 2021 Dec 6;11:23447. doi: 10.1038/s41598-021-02619-y (PMC8648872; doi:10.1038/s41598-021-02619-y)
Supplement: Supplementary file 1 — Supplementary Information. [file 41598_2021_2619_MOESM1_ESM.pdf]

A Host-Directed Macrocyclic Peptide Therapeutic for MDR Gram Negative Bacterial Infections

Justin B. Schaal*, Yoshihiro Eriguchi, Dat Q. Tran, Patti A. Tran, Chase Hawes, Anthony Cabebe, Kaitlyn Pike, Katie Trinh, André J. Ouellette, Michael E. Selsted

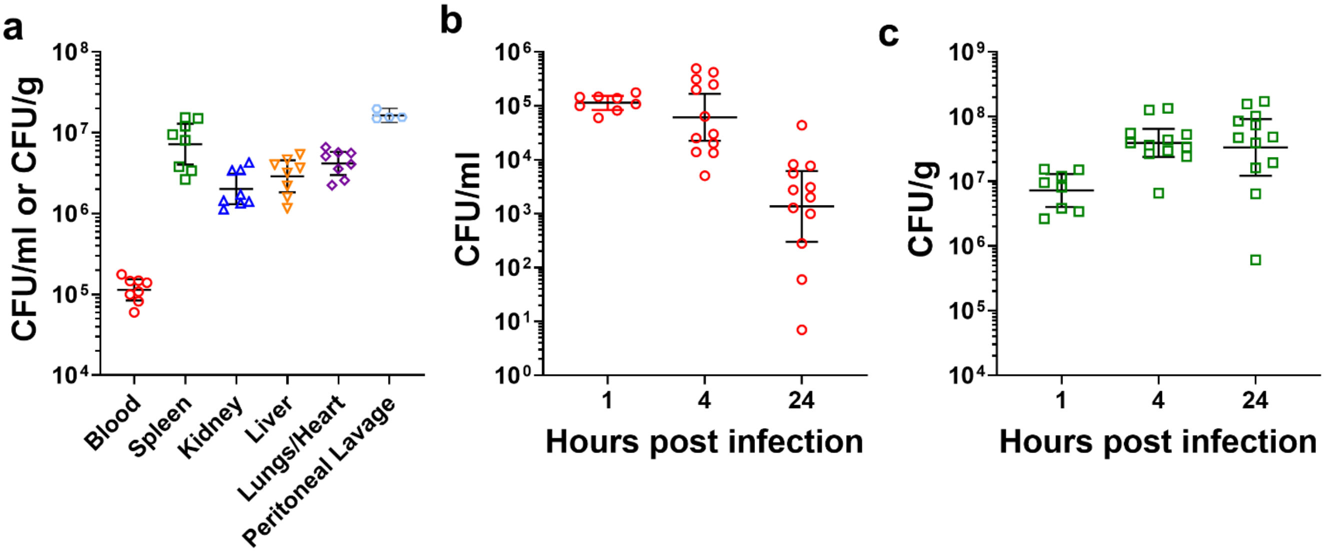

Supplemental Figure 1: Rapid dissemination of *Kp*-1705 following intraperitoneal infection. (**a-c**) Male and female BALB/c mice were challenged i.p. with *Kp*-1705 (3 - 5 x 10^8 CFU). (**a**) One h p.i. animals were euthanized and bacterial burden determined for blood, spleen, kidney, liver, lungs/heart, and peritoneal lavage (n=8). Bacterial burden in blood (**b**) and spleen homogenate (**c**) were determined at 1, 4 and 24 h p.i. Data are means ± 95% CI.

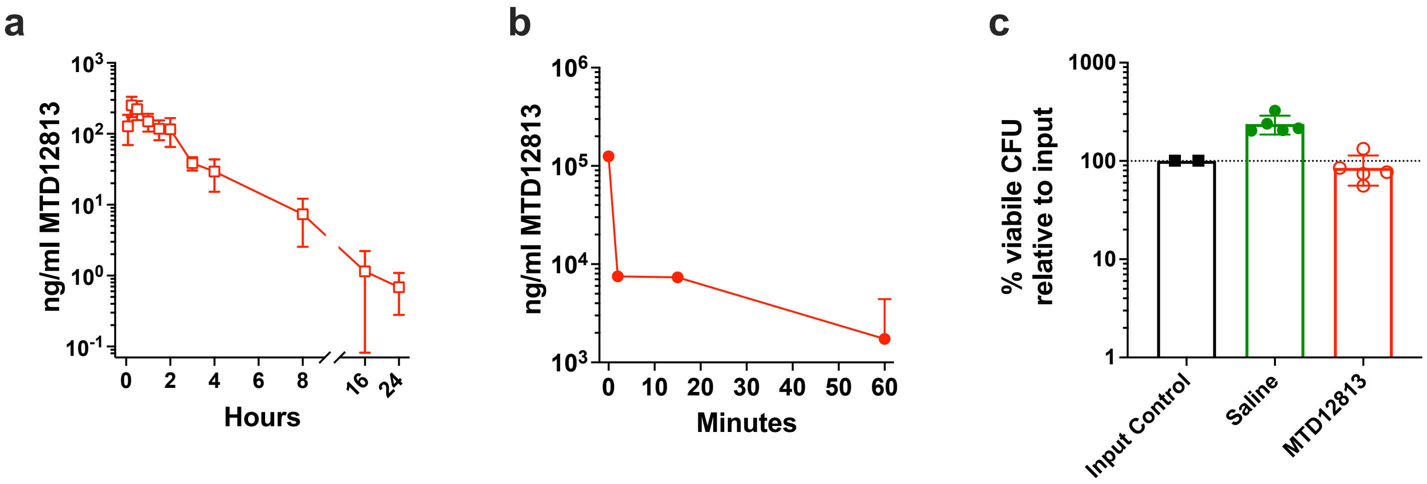

Supplemental Figure 2. MTD12813 pharmacokinetics and peritoneal fluid antimicrobial activities. (**a-b**) Naïve BALB/c mice (2M/2F per time point) received 1.25 mg/kg MTD12813 by a single i.p. injection. (**a**) Blood plasma and (**b**) peritoneal fluid peptide levels were quantified by LC-MS/MS. **(c)** Peritoneal fluid was collected from uninfected mice (2M/2F) injected i.p. with saline or 1.25 mg/kg MTD12813. Fluids were inoculated *in vitro* with 2 x 10^7 CFU/ml of *Kp*-1705, incubated for 1 h at 37° C, and viability determined by counting CFU on TSA plates.

Cytokine	Hour p.i.	Sham (n=5) Mean	SD	Saline (n=11-12) Mean	SD	MTD12813 (n=11-12) Mean	SD	Delta	% Change	P-value
**IL-6**	2			266274.4	± 149491.2	216618.0	± 97937.0	-49656.5	-18.6	0.472
	4	24.5	± 20.0	239682.6	± 150080.3	128253.4	± 109127.5	-111429.2	-46.5	**0.0328**
	24			224517.4	± 278673.0	687.0	± 481.4	-223830.4	-99.7	**4.38×10^{-6}**
**CXCL1 (KC)**	2			364564.4	± 167803.7	258876.9	± 142590.2	-105687.5	-29.0	0.1260
	4	169.7	± 113.2	380278.4	± 305680.2	168047.7	± 111004.5	-212230.7	-55.8	**9.59×10^{-3}**
	24			118116.7	± 143173.3	1365.9	± 854.1	-116750.8	-98.8	**1.25×10^{-5}**
**CXCL10 (IP-10)**	2			29029.4	± 6754.2	37616.2	± 12095.1	8586.9	29.6	0.126
	4	200.1	± 58.0	110999.9	± 40362.5	68204.6	± 15429.1	-42795.3	-38.6	**2.45×10^{-3}**
	24			9666.3	± 9142.1	1667.5	± 229.7	-7998.8	-82.7	**3.39×10^{-4}**
**CCL2 (MCP-1)**	2			62216.0	± 7150.9	59107.8	± 9072.5	-3108.2	-5.0	0.462
	4	48.8	± 50.7	60693.2	± 11269.2	33486.3	± 10505.8	-27206.9	-44.8	**2.68×10^{-5}**
	24			53241.7	± 94318.5	664.5	± 313.4	-52577.2	-98.8	**8.57×10^{-5}**
**CCL4 (MIP-1β)**	2			64892.4	± 9360.0	46231.0	± 13225.6	-18661.3	-28.8	**9.30×10^{-3}**
	4	16.3	± 9.3	35052.8	± 7096.8	24260.1	± 5045.1	-10792.7	-30.8	**7.45×10^{-4}**
	24			3674.5	± 4025.7	409.9	± 163.9	-3264.6	-88.8	**1.66×10^{-4}**
**CXCL2 (MIP-2)**	2			145483.3	± 23309.5	105959.4	± 38123.4	-39523.9	-27.2	**0.0379**
	4	208.3	± 125.0	51686.3	± 23415.4	27003.7	± 18839.5	-24682.6	-47.8	**0.0117**
	24			53979.5	± 70550.0	228.1	± 86.3	-53751.4	-99.6	**3.71×10^{-5}**
**CCL11 (Eotaxin)**	4	917.8	± 452.8	3962.8	± 589.1	3978.9	± 435.1	16.1	0.4	0.865
	24			5046.0	± 2719.7	1258.5	± 296.0	-3787.5	-75.1	**3.24×10^{-6}**
**IFNγ**	4	1.1	± 0.0	77.7	± 48.5	57.5	± 47.9	-20.2	-26.0	0.246
	24			109.6	± 144.9	1.1	± 0.0	-108.5	-99.0	**9.45×10^{-4}**
**IL-1α**	4	67.5	± 112.1	277.9	± 80.6	282.8	± 103.6	4.9	1.8	0.973
	24		±	1030.9	± 1915.3	93.7	± 57.8	-937.2	-90.9	**4.89×10^{-3}**
**IL-1β**	4	5.4	± 0.0	123.7	± 62.2	104.9	± 60.0	-18.8	-15.2	0.548
	24			177.9	± 267.3	5.2	± 1.3	-172.6	-97.1	**0.0288**
**IL-5**	4	1.0	± 0.0	154.6	± 131.5	189.3	± 138.1	34.6	22.4	0.294
	24			485.2	± 629.9	16.2	± 21.2	-469.0	-96.7	**7.07×10^{-4}**
**IL-9**	4	126.5	± 197.2	236.4	± 139.2	491.8	± 298.9	255.4	108.0	**0.032**
	24			695.5	± 709.8	49.9	± 54.1	-645.5	-92.8	**7.30×10^{-3}**
**IL-10**	4	2.0	± 0.0	1472.0	± 417.1	1768.1	± 505.6	296.1	20.1	0.196
	24			4275.1	± 3340.0	296.6	± 216.3	-3978.5	-93.1	**4.57×10^{-5}**
**IL-12 (p40)**	4	3.9	± 0.0	166.0	± 79.8	137.7	± 34.3	-28.3	-17.0	0.504
	24			25.6	± 23.7	3.9	± 0.0	-21.7	-84.7	**3.78×10^{-3}**
**IL-12 (p70)**	4	4.8	± 0.0	180.8	± 42.1	200.0	± 67.6	19.1	10.6	0.650
	24			129.4	± 110.4	4.8	± 0.0	-124.6	-96.3	**7.50×10^{-4}**
**LIF**	4	1.0	± 0.0	37.1	± 15.8	28.6	± 22.8	-8.5	-22.9	0.108
	24			514.1	± 677.1	1.0	± 0.0	-513.1	-99.8	**1.59×10^{-6}**
**IL-13**	4	103.7	± 23.8	276.2	± 54.4	311.0	± 71.2	34.8	12.6	0.302
	24			211.9	± 118.2	25.7	± 16.0	-186.2	-87.9	**7.21×10^{-7}**
**CXCL5 (LIX)**	4	1581.1	± 715.6	2830.2	± 1294.5	3115.6	± 2014.5	285.4	10.1	0.981
	24			4240.1	± 2475.4	678.2	± 303.6	-3561.9	-84.0	**2.62×10^{-6}**
**IL-15**	4	124.9	± 92.8	382.9	± 161.3	642.5	± 276.2	259.7	67.8	**9.14×10^{-3}**
	24			662.1	± 335.2	391.3	± 355.3	-270.9	-40.9	0.0519
**IL-17**	4	2.2	± 3.4	46.4	± 21.5	66.4	± 27.4	19.9	42.9	0.111
	24			1613.4	± 2215.2	0.8	± 0.7	-1612.6	-100.0	**1.06×10^{-7}**
**CCL3 (MIP-1α)**	4	46.1	± 44.1	1446.7	± 625.9	866.2	± 318.7	-580.5	-40.1	**5.72×10^{-3}**
	24			595.6	± 381.8	161.8	± 58.9	-433.8	-72.8	**6.84×10^{-5}**
**M-CSF**	4	3.5	± 0.0	122.8	± 60.3	118.1	± 50.1	-4.8	-3.9	0.947
	24			175.4	± 219.1	7.8	± 6.4	-167.6	-95.5	**8.88×10^{-3}**
**GM-CSF**	4	10.9	± 0.0	84.5	± 16.2	75.2	± 24.8	-9.2	-10.9	0.282
	24			102.9	± 109.8	10.9	± 0.0	-92.0	-89.4	**1.15×10^{-3}**
**CXCL9 (MIG)**	4	34.8	± 29.1	4108.2	± 476.4	3811.4	± 778.2	-296.8	-7.2	0.265
	24			4839.0	± 3354.1	873.1	± 208.2	-3965.9	-82.0	**1.61×10^{-5}**
**CCL5 (RANTES)**	4	3.9	± 2.4	1205.2	± 314.6	722.8	± 390.1	-482.4	-40.0	**0.0166**
	24			1003.9	± 680.1	118.3	± 42.0	-885.6	-88.2	**6.57×10^{-6}**
**TNF**	4	2.3	± 0.0	241.2	± 56.7	166.6	± 30.3	-74.6	-30.9	**9.33×10^{-4}**
	24			214.3	± 190.9	21.9	± 7.0	-192.4	-89.8	**3.53×10^{-5}**

Supplemental Table 1. Mice were challenged i.p. with *Kp*-1705 and treated 1 h later with MTD12813 (1.25 mg/kg i.p.) or saline. The sham cohort received bacteria-free suspension buffer at t=0 followed by saline 1 h later. Mice were euthanized 2, 4, or 24 h p.i., and plasma samples were subjected to multiplex cytokine analysis. P values calculated using ANOVA with Uncorrected Fisher's LSD comparing saline and MTD12813 treated mice at respective time points.

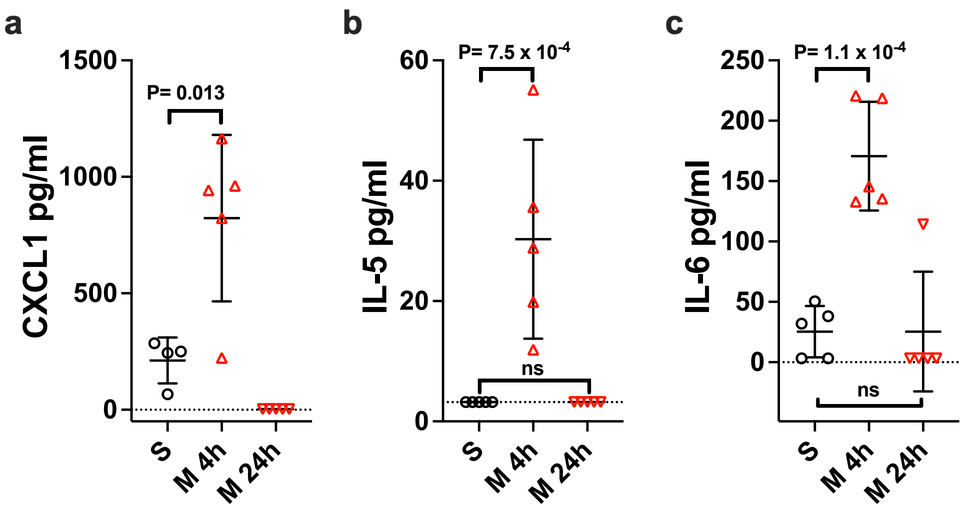

Supplemental Fig. 3. Transient stimulation of cytokines by MTD12813. Sham infected mice were injected i.p with PBS and treated i.p. 1 h later with 1.25 mg/kg MTD12813 (M) or saline (S). Four or 24 h post sham infection, animals were euthanized and plasma samples quantified by multiplex cytokine analysis. P values calculated using ANOVA with Uncorrected Fisher's LSD comparing saline and MTD12813 treated mice.

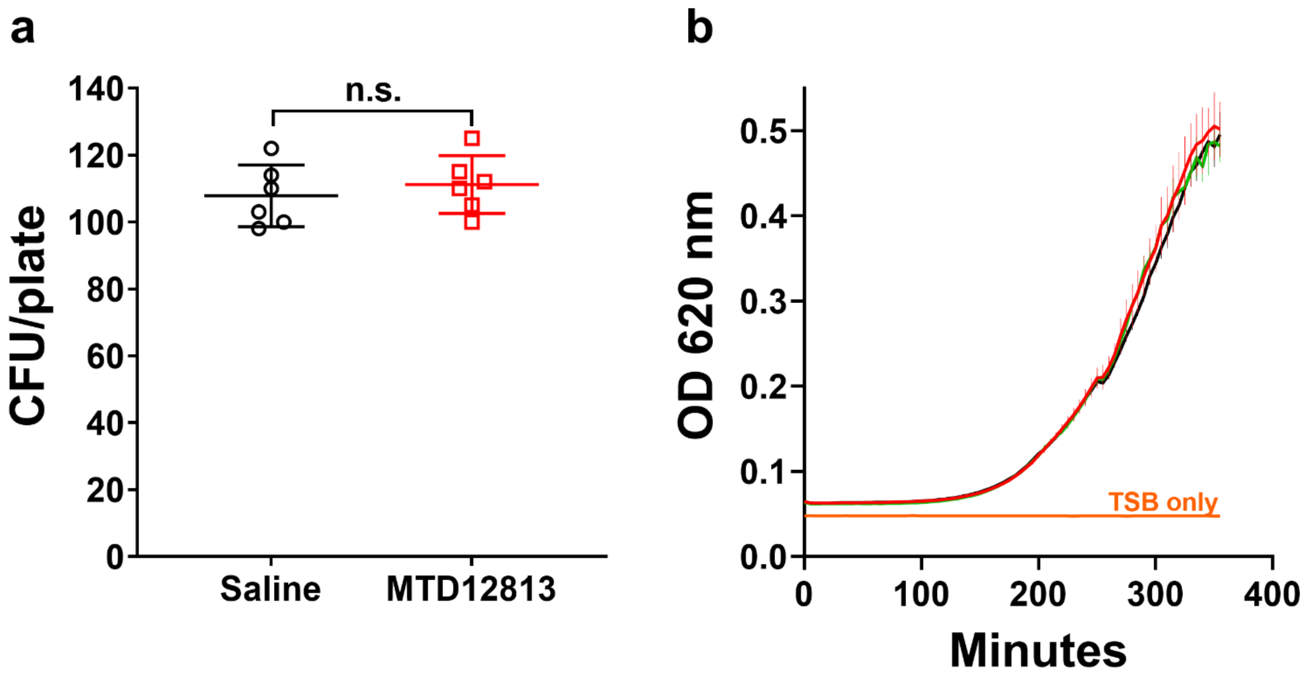

Supplemental Fig 4. Sub-MIC concentrations of MTD12813 does not affect bacterial viability or replication fitness of *Kp*-1705. *Kp*-1705 (1 x 10^9 CFU/ml) was incubated for 1 h with 1.25 µg/ml MTD12813 or saline. (**a**) Bacterial viability was quantified as CFU on TSA plates. P value determined by Student's t-test. (**b**) *Kp*-1705 replication fitness was determined by incubating a 40-fold dilution of the incubation mixtures from panel (**a)** in TSB and measuring bacterial growth (black-saline; red-MTD12813) at A_{620}. The green growth curve is of bacteria incubated with TSB in the presence of 1.25 µg/ml MTD12813 and "TSB only" is absorbance of sterile medium. Samples were analyzed in triplicate with standard deviation shown.
